# PHOG: a database of supergenomes built from proteome complements

**DOI:** 10.1186/1471-2148-6-52

**Published:** 2006-06-22

**Authors:** Igor V Merkeev, Pavel S Novichkov, Andrey A Mironov

**Affiliations:** 1 State Scientific Center GosNIIGenetica, 1st Dorozhny pr., 1, Moscow, 113545, Russia; 2 National Center for Biotechnology Information, U.S. National Library of Medicine, 8600 Rockville Pike, Bethesda, MD 20894, USA; 3 Department of Bioengineering and Bioinformatics, Moscow State University, Vorob'evy gory, 1–73, Moscow, 119992, Russia

## Abstract

**Background:**

Orthologs and paralogs are widely used terms in modern comparative genomics. Existing procedures for resolving orthologous/paralogous relationships are often based on manual revision of clusters of orthologous groups and/or lack any rigorous evolutionary base.

**Description:**

We developed a completely automated procedure that creates clusters of orthologous groups at each node of the taxonomy tree (PHOGs – Phylogenetic Orthologous Groups). As a result of this procedure, a tree of orthologous groups was obtained. Each cluster is a "supergene" and it is represented by an "ancestral" sequence obtained from the multiple alignment of orthologous and paralogous genes.

The procedure has been applied to the taxonomy tree of organisms from all three domains of life. Protein complements from 50 bacterial, archaeal and eukaryotic species were used to create PHOGs at all tree nodes. 51367 PHOGs were obtained at the root node.

**Conclusion:**

The PHOG database demonstrates that it is possible to automatically process any number of sequenced genomes and to reconstruct orthologous and paralogous relationships between genomes using a rigorous evolutionary approach. This database can become a very useful tool in various areas of comparative genomics.

## Background

Evolutionary forces acting on genomes result in gene duplications, gene losses and gene acquisitions. Generally, it is difficult to reconstruct the exact evolutionary history of a protein family due to its complex nature. A widely used approach to study such history is to find orthologous groups by comparing completely sequenced genomes. This approach resulted in several databases [1-4] that helped to predict protein function and provided deep insights into the protein evolution. These procedures, however, did not fully take into account the taxonomy tree of organisms.

Orthologs are genes derived from a single ancestral gene as a result of the speciation event, while paralogs are genes that result from gene duplication events [5-7]. The usefulness of orthologs and paralogs in modern genomics comes from the fact that the products of orthologs generally perform the same function while the products of paralogs perform a similar function. We can give several examples how the knowledge of orthologs and paralogs helped to solve some difficult issues. Comparative studies of bacterial transcriptional regulation often use orthologs assuming that orthologs tend to be regulated in the same way [8-10]. It is possible to predict functional coupling between genes if orthologs of genes forming a functional cluster in one organism will form a cluster in another organism [11]. Leonid Mirny and Mikhail Gelfand [12] have found specificity-determining positions in the LacI/PuR family of bacterial transcription factors looking for residues that are conserved among orthologs and are different in paralogs. Orthologs and paralogs also help to understand the evolution by gene duplication, which is thought to be a major force in creating organismal complexity [13,14]. If clusters of orthologous groups are found that contain mainly genes from a particular group of organisms [15,16], it is possible to better understand physiology specific for this group of organisms.

**Figure 1:**
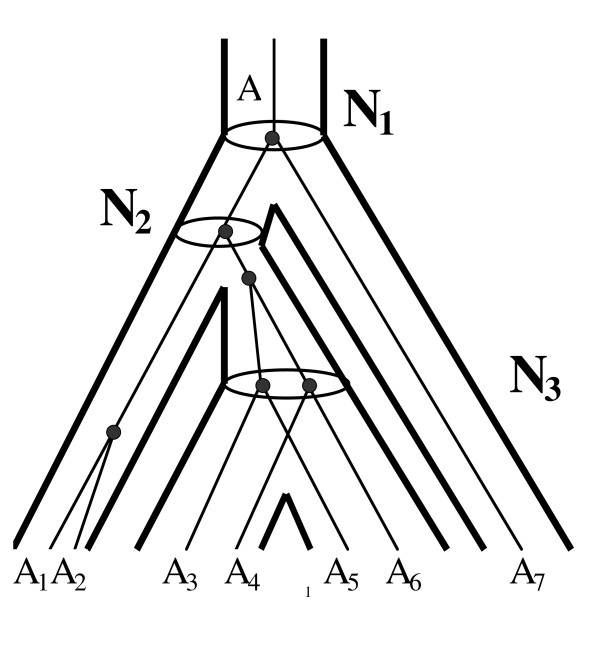
Evolution by gene duplication. Nodes N_1_, N_2_, N_3 _represent speciation events resulting in orthologs. Filled circles (●) mark gene duplication events resulting in paralogs.

Fig. 1 shows what issues might arise where resolving the orthologous/paralogous relationships between genes. An ancestral gene A creates a family of genes A_1_, A_2_, A_3_, A_4_, A_5_, A_6_, A_7 _by three speciation events N_1_, N_1_, N_3 _and two gene duplication events. The real evolution of gene families is far more complex than this simple example creating a complex network of orthologs and paralogs. A gene is considered to be an ortholog or a paralog relative to a particular node N of the evolutionary tree if its ancestor at the child node following the node N is a result of a speciation event or a gene duplication event correspondingly. For instance, the gene A_3 _is an ortholog to the gene A_5 _since they both are the result of the speciation event occurred at the node N_3_, while this gene is a paralog to the gene A_1_because it is the result of a gene duplication event occurred after the speciation event at the node N_2_. How can we resolve these relationships for hundreds of organisms having thousands of genes? To correctly resolve orthologs and paralogs, we suggest that clusters of orthologous genes should be defined at each node of the taxonomy tree of organisms. Indeed, if such clusters are obtained for the tree in Fig. 1, then it will be clearer how to reconstruct the evolutionary history of the protein family A. At the node N_3, _the genes A_3_, A_5 _will form one independent orthologous group since they were derived from some ancestral gene A_35_, and the genes A_4_, A_6 _will form another independent orthologous group since they were derived from some ancestral gene A_46_. We can consider the pairwise alignment built form A_3 _and A_5 _as a representative of their ancestral gene A_35. _The same is true for the genes A_4 _and A_6_. Extending this idea of grouping genes to represent their ancestors, we can say that at the node N_2 _the genes A_1_, A_2_, A_3_, A_4_, A_5 _and A_6 _will form their own independent orthologous group. In this orthologous group the gene A_1 _and the orthologous group (A_4_, A_6_) from the node N_3 _will be orthologs, and the gene A_2 _and the orthologous group (A_3_, A_5_) from the node N_3_will be paralogs.

Our procedure is based on the direct definition of orthologs and paralogs and utilizes the following idea. If we have several species with their proteomes at one node of the taxonomy tree of organims, we can find orthologs by running a similarity search procedure (e.g. BLAST) between each pair of species, find bi-directional best hits (BBHs), and choose orthologs from BBHs using some system of rules. Then it is possible to find paralogs in each species by finding genes that are not declared orthologs and which have the statistically significant best hit to an already found orthologous group. Then we can form a new "genome", putting into it all orthologous families and genes that did not find any match. Since this new "genome" is an artificial construct and it includes all genes from both species, this new genome is called a supergenome built from protein complements of both species. In the same way, we can also find orthologs and paralogs between two supergenomes and build a next level supergenome. Repeating the procedure for all nodes of the tree, we will eventually obtain the root level supergenome. Since clusters of orthologous groups are defined at each node of the taxonomy tree, they are called PHOGs (Phylogenetic Orthologous Groups). A supergenome is a collection of PHOGs accumulated at a particular node of the taxonomy tree. A supergene is an "ancestral" sequence for a PHOG.

There are four fundamental differences between our procedure and the earlier procedures to obtain clusters of orthologous groups [1-4]: (i) our procedure is completely automated, so it does not require any manual intervention; (ii) our procedure uses evolutionary approach to detect orthologs and paralogs; (iii) our procedure creates clusters of orthologous groups at each node of the evolutionary tree and gives clear indication of the timing of gene duplication events that result in paralogs; (iv) the time required to run our procedure depends linearly on the number of genomes.

## Construction and content

The basic step in the overall procedure to obtain PHOGs at all nodes of the evolutionary tree is to compare several supergenomes, find orthologs and paralogs, put them into one PHOG and to merge these supergenomes into the supergenome lying higher in the evolutionary tree. Since each PHOG represents a multiple alignment of protein sequences, it has first to be converted into an "ancestral" sequence (a supergene), and then consensus sequences from both supergenomes are compared to find orthologs and paralogs. Sequences in each newly created PHOG are multiply aligned, and all PHOGs are then stored in the relational database to launch the procedure at next nodes of the evolutionary tree.

### Obtaining a supergene from a PHOG multiple alignment

Our accompanying paper [17] shows that each column of the multiple alignment in more than 98% cases belongs to one of the 20 frequency column clusters, which can be thought to be derived from a single amino acid residue. Rarely, we obtain "garbage' columns which will get the special symbol "X". We convert a column of the protein multiple alignment to a frequency vector and find the nearest cluster as described in our paper [17].

### Running PHOG-BLAST

After all PHOGs from supergenomes are converted into consensus sequences, a special BLAST-like procedure is run between each pair of supergenomes lying at a single node of the evolutionary tree which is called PHOG-BLAST [17]. PHOG-BLAST combines ideas from FASTA [18], original BLAST [19] and dynamic programming.

**Figure 2:**
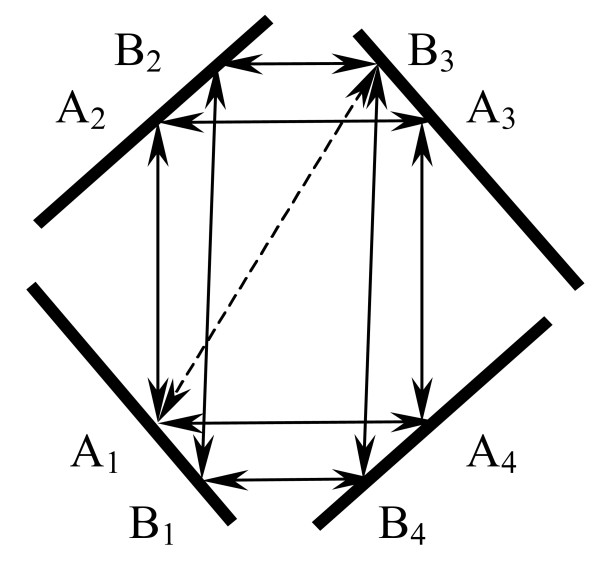
One connected component contains two orthologous groups A_1_A_2_A_3_A_4 _and B_1_B_2_B_3_B_4. _The false BBH bridge A_1_B_3 _connects both orthologous groups.

**Figure 3:**
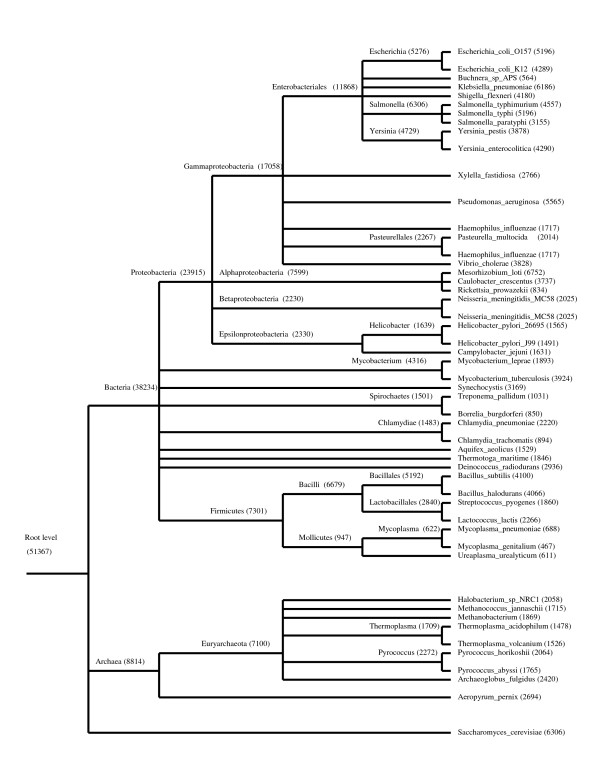
The taxonomy tree of organisms used to build the PHOG database. The number of PHOGs at each node of the tree is shown in parentheses.

After PHOG-BLAST scores are computed for all possible pairs of supergenes from each pair of supergenomes, bi-directional best hits are obtained (BBHs). Since BBHs with low scores can potentially lead to false-positive orthologs, BBHs with scores less than a given threshold (100) are discarded. If we form a graph with vertices as supergenes and BBH relationships as edges we can obtain connected components in this graph. Fig. 2 shows a connected component in this graph consisting of eight genes. Genes A_1_, A_2_, A_3_, A_4 _form one orthologous group and genes B_1_, B_2_, B_3_, B_4 _form another orthologous group. Due to the false BBH bridge between genes A_1 _and B_3_, both orthologous groups are merged into a single orthologous group. Some of these connected components can be quite big. For example, when our procedure was run at the Archaen node (Fig. 3), we frequently obtained connected components having 60 vertices and more. Since we believe that each orthologous group is the result of the node evolution of just one ancestral gene we have to split this connected component into several parts.

### Splitting procedure

Our splitting procedure is based on the assumption that the higher the BBH score is between a pair of supergenes, the greater is the chance that these BBH supergenes are orthologs and they are not false BBH bridges. Therefore, we are looking for the pair of supergenes in the connected component with the greatest BBH score, and we consider this pair as the seed of a new orthologous group. For all other supergenes in the connected components we calculate a sum of PHOG-BLAST scores to the seeds. Then we arrange these supergenes in descending order for these scores. After that we "fill" the growing orthologous group starting from the top ranking genes in this order and omitting genes that have already representatives in the orthologous group from their taxon. We repeat this procedure for all genes that are not included in orthologous groups until we cannot find seeds anymore. As a result of this procedure, the connected component is split into several orthologous groups, each with its pair of seed supergenes. To reduce the rate of erroneous assignment of supergenes to orthologous groups, we reshuffle all supergenes assigning them to those seeds for which they have the maximum PHOG-BLAST score.

The possible scenario for our procedure for the situation in Fig. 2 might be like this. A_1_A_2 _is the strongest BBH, the arrangement of other supergenes is A_3_A_4_B_1_B_2_B_3_B_4_, and the first "filled" orthologous group is A_1_A_2_A_3_A_4_. From all BBHs that are not included in this orthologous group, B_1_B_2 _is the strongest BBH, the arrangement of other supergenes is B_3_B_4_. The second "filled" orthologous group is B_1_B_2_B_3_B_4_. Reshuffling has not changed the composition of both orthologous groups.

In each orthologous group, the Smith-Waterman algorithm [20] is applied to the seeds. Only seed segments giving the maximum score are left for further processing. They are called seed cores. A seed consensus sequence is formed from these cores by finding the nearest frequency profile cluster in each position of this seed pairwise alignment. Since N/C out-of-core ends of seeds might represent protein domains, it is very important to look at them once more. To this end, N/C out-of-core ends having length greater than 100 are stored in the database and they are used to launch the second round of the procedure at a single node of the evolutionary tree (see the "Second round" section below).

Similarly, the Smith-Waterman algorithm is applied to the seed consensus and all non-seed supergenes to get non-seed cores. N/C out-of-core ends of non-seeds having length greater than 100 are stored in the database.

### Multiple alignment of core sequences in the orthologous group

In our earlier experiments with PHOGs we used ClustalW [21] to multiply align supergene sequences. However, this approach resulted in a very slow overall procedure. Therefore, we decided to develop our own procedure for the multiple alignment, following the traditional iterative approach. The computational experiments showed that this procedure produced multiple alignments of good quality (data not shown).

Our alignment procedure is based on the well-known observation that more similar protein sequences produce less error prone alignments [22]. The input for our multiple alignment procedure is a set of supergene core sequences belonging to one orthologous group obtained at the previous step. The procedure runs as follows:

(i) Compute a sum of PHOG-BLAST scores for non-seed cores to the seed cores.

(ii) Arrange all core sequences in the descending order for these scores. Seed cores will head the ordered list.

(iii) Set the consensus sequence equal to the first sequence in the ordered list.

(iv) Set the current sequence equal to the second sequence in the ordered list.

(v) Apply the Needleman-Wunsch algorithm [23] to align the consensus sequence and the current core sequence. Form the new consensus sequence from this multiple alignment of the two aligned sequences by finding the nearest frequency column cluster in each position.

3. Repeat step (v) for all other sequences in the ordered list.

### Finding paralogs

After gene duplications, paralogs experience a period of relaxed evolution [14], and generally it is difficult to assess how long this period was. One safe approach to find paralogs is to select those gene as paralogs whose evolutionary distance to an ortholog in its own taxon is smaller than the distance between orthologs belonging to different taxons [24]. We think, however, that this approach is too restrictive, and the procedure based on it can result in too many orphan genes, even these genes have high similarities to other genes that found their counterparts in other species and fell into PHOGs.

Therefore, for all supergenes that are not declared orthologs at a particular node of the evolutionary tree, we compute PHOG-BLAST scores to PHOG supergenes and for each such supergene we find the best hit. If the PHOG-BLAST score to this consensus exceeds 100, we declare this supergene to be a paralog to the best-hit PHOG. After all paralogs are added to PHOGs, PHOGs are aligned as described in the previous section.

### Second round of the procedure

This round is needed because orthologs can have different domain structures due to gene fusion events. If both orthologs have a homologous core, but the first ortholog has an additional domain that is absent in the second ortholog, then we can cut out this additional domain. This additional domain can find its match among other domains or orphan genes in other supergenomes. Therefore, all previous steps are repeated for all N/C cuts and all orphan genes at a particular node.

## Utility

The PHOG database can be used in various areas of comparative genomics, such as studying the evolution of protein function, finding proteins specific to a particular group of organisms, determining protein fusions and protein domain structure, functional annotation of sequenced genomes.

## Discussion

The procedure has been applied to the tree of organisms shown at Fig. 3. Proteomes for these species were downloaded from the NCBI ftp site [25]. 51367 PHOGs were obtained at the root node of the tree including 36903 PHOGs that consisted of only one gene (orphan genes). 14464 PHOGs contained at least two protein sequences. Table 1 shows several key values for the nodes leading from *Escherichia coli *O157 to the Universal Common Ancestor. As we move from lower nodes to upper nodes, the number of PHOGs per node sharply rises, while the number of ancestral PHOGs is stable within the range from 2000 to 5000 PHOGs. At any node, ancestral PHOGs form a subset of all PHOGs available at that node. By definition, they are PHOGs that contain two or more PHOGs from its child nodes that were declared as orthologs and possibly some PHOGs from its child nodes that were declared as paralogs. The supergenes for these PHOGs can be thought as ancestral genes belonging to some hypothetical ancestral organism that gave rise to all taxonomy groups lying lower in the taxonomy tree. Node-specific PHOGs consist of all ancestral PHOGs that did not find their match during the procedure run for all nodes lying higher in the taxonomy tree.

**Table 1:** Number of PHOGs obtained at the nodes of the taxonomy tree for the lineage leading from the Universal Common Ancestor to *Escherichia coli *O157. For each node, ancestral PHOGs (N_a_) contain two or more PHOGs from its child nodes that were declared as orthologs and possibly some PHOGs from child nodes that were declared as paralogs (N_p_). Ratio N_p_/N_a _indicates how many paralogs evolved from N_a _ancestral genes. Node-specific PHOGs (N_ns_) consist of all ancestral PHOGs that did not find their match during the procedure run for all nodes lying higher in the taxonomy tree.

**Node of the taxonomy tree**	**Escherichia coli O157**	**Escherichia**	**Enterobacteriales**	**Gamma-proteobacteria**	**Proteobacteria**	**Bacteria**	**Universal common ancestor**
Total number of PHOGs, N	5196	5276	11868	17058	23915	38234	51367
Number of node-specific PHOGs, N_ns_	578	161	1327	934	996	2079	2055
Number of ancestral PHOGs, N_a_	5196	3780	5190	3766	3104	3827	2055
Number of paralogs, N_p_	0	629	3101	2373	1576	2453	1620
N_p_/N_a_	0	0.166	0.597	0.63	0.507	0.64	0.788

All ancestral PHOGs that are not node-specific could be considered as a result of vertical evolution from some PHOGs lying higher in the taxonomy tree. The evolution of node-specific PHOGs is an evolutionary mystery. For the nodes corresponding to currently living organisms, node-specific PHOGs are usually called orphan gene. Tomislav Domazet-Loso and Diethard Tautz [26] give three reasons why a gene can become orphan: (i) the gene is newly evolved; (ii) the gene was lost in most evolutionary lineages; (iii) the gene evolves very quickly. The ratio of the number of paralogs to the number of ancestral PHOGs (N_p_/N_a_) is within the range from 0.15 to 0.8 suggesting that gene duplications and gene losses probably played a major role in the evolution of life. During the early stages of evolution of Life on Earth gene duplications and the formation of node-specific genes happened on a larger scale than during the later stages of evolution. Take, for example two nodes: Bacteria and Escherichia. For bacteria N_p_/N_a _is 0.64, whereas for Escherichia this ratio is only 0.166. The number of node-specific PHOGs is 2079 for Bacteria and only 161 for Escherichia. Clearly, to create a new taxon such as Bacteria nature had to evolve more new genes than to create such a taxon as Escherichia from a closely related ancestral taxon.

We used the COG database [27] to test our procedure as the most complete database of orthologous group available today. Since this database also contains protein sequences for most organisms from our tree, we matched protein sequences in our database against the COG database. Each matched protein sequence obtained a number corresponding to the number of the COG where this sequence was found. 14464 non-orphan PHOGs contained 83450 thus matched protein sequences. Each such PHOG obtained a COG number corresponding to the biggest number of protein sequences from this COG in this PHOG. Sequences whose COG numbers were different from COG numbers of their PHOGs were counted with the total count of 2472. Thus, we obtained the mismatch rate about 3%. This test proves that our procedure basically results in the same clusters of orthologous groups, though the composition of corresponding COGs and PHOGs can be somewhat different due to the great amount of statistical material and ambiguities of the evolution of protein families. For each COG there is a corresponding PHOG. The number of PHOGs is, however, five times greater than the number of COGs in the COG database. There are two main reasons for that. First, COGs that contain fusion proteins are split into several PHOGs containing their domains. Second, since the procedure that underlies the COG database [1] uses the triangle pattern of BBHs, it might not include clusters of orthologous groups that arise at lower level of the taxonomy tree. For example, at the Escherichia coli node of the taxonomy tree we detected 432 orphan PHOGs that contained genes only from two closely related strains: *Escherichia coli *O157 and *Escherichia coli *K12. Obviously, these PHOGs did not have their counterparts in the COG database.

**Figure 4:**
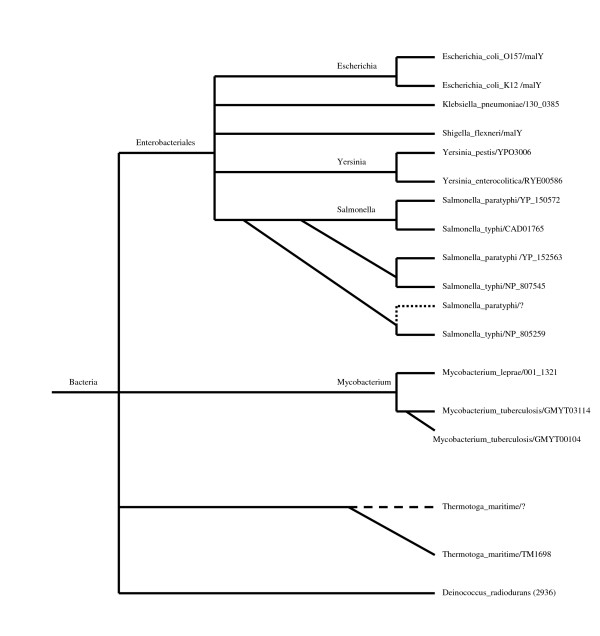
A possible evolutionary scenario for the PHOG16006. Dashed lines indicates gene losses.

The PHOG database provides a possible evolutionary scenario for the evolution of a particular PHOG. If a PHOG consists of orthologs only, then we do need to care about gene duplications. If a PHOG contains paralogs, the PHOG database indicates the probable timing interval of gene duplication events for all PHOGs that contain paralogs. Consider, for example, PHOG16006. Its possible evolutionary scenario is shown in Fig. 4. The gene TM1698 from *Thermotoga maritima *is a paralog at the Bacteria node of the taxonomy tree. Therefore, it is, possibly, the result of a gene duplication event occurred anywhere during the evolutionary process from the ancestor of Bacteria to modern *Thermotoga maritima*. Since PHOG16006 does not contain an ortholog from *Thermotoga maritima *at the Bacteria node, we can assume that this gene was lost. The gene YP_152563 from *Salmonella paratyphi *and the gene NP_807545 from *Salmonella typhi *(putative aminotransferases) form an orthologous group at the Salmonella node of the taxonomy tree. At the Enterobacteriales node, this orthologous group becomes a paralog, with a duplication event occurred somewhere between the Enterobacteriales node and the Salmonella node. The gene NP_805259 (putative aminotransferase) of *Salmonella typhi *is also a paralog at the Enterobacteriales node, but it lacks its ortholog at the Salmonella node, because it was probably lost.

The average length of the root level supergene is 310 amino acids, which corresponds approximately to two protein domains. As the procedure goes from the leaves to the root of the evolutionary tree, protein sequences are truncated to leave the most conserved evolutionary cores. There is always a possibility that cores can be truncated to such extent that they cannot be used anymore for resolving orthologs and paralogs. The remarkable fact about the root level PHOGs is that their lengths are not seriously shortened. This observation leads us to the startling proposal that the core determine the general function for the protein family in one PHOG and protein N/C ends determine species-specific behavior.

An interesting feature of the PHOG database is that it provides a built-in capability to detect fusion events and the multidomain structure of proteins due to its N/C cuts each time when the domain structure of orthologous groups is different. Therefore, it will be more correct to call the PHOG database as a database of orthologous domains. We can give several examples. COG1526 is split into two PHOGs: PHOG722 and PHOG51085. These two PHOGs contain possible domains of fusion proteins that exist as single entities in the COG database. For example, the gene VC1519 of *Vibrio cholera *is only included in COG1526, whereas in the PHOG database its possible domains are present in PHOG722 and PHOG51085. We also detected fusion proteins for COG1217 (it is split into PHOG30 and PHOG34) and COG60 (it is split into PHOG39 and PHOG50466). A striking example is COG1674. It is split into six PHOGs. The protein BH0975 (unknown conserved protein) from *Bacillus halodurans *is present in all these PHOGs indicating that it consists of at least 6 domains, while it is present only in COG1674 in the COG database. We used the CDD database [28] to verify the domain structure of BH0975. This database refers to four conserved domains: CDD:11385 (DNA segregation ATPase), CDD:25783 (putative ATP binding P-loop motif), CDD:25783 (putative ATP binding P-loop motif) and CDD:25783 (putative ATP binding P-loop motif). The PHOG database finds two additional putative domains at the N/C ends of the protein. These domains are conserved only between two closely related species: *Bacillus halodurans *and *Bacillus subtilis*. This observation also supports the idea that protein N/C ends determine species-specific behavior.

We used the NCBI taxonomy tree [29] as the tree that controls the flow of our procedure from the leaves of the tree to its root. No one tree can be absolutely perfect, and there can be ambiguities and errors in assigning organisms to taxonomy groups. When changing the assignment of a particular organism to a different node of the tree, the composition of the PHOGs at affected lower levels of the tree will be slightly different reflecting this new assignment. However, at higher nodes of the tree the composition of PHOGs will be the same, since BBH relationships undiscovered at lower nodes will be rediscovered at higher nodes. We can give the following example. Earlier we mentioned 432 orphan PHOGs detected at the Escherichia coli node of the taxonomy tree. If we move *Escherichia coli *O157 to another node, say the Salmonella node, then these 432 orphan PHOGs will be rediscovered at the Enterobacteriales node of tree.

## Conclusion

The computer procedure that was used to build the PHOG database can take any number of sequences genomes with predicted protein sequences to build orthologous groups. This opens new vistas for studying protein evolution. Using this database the researcher can compare not only proteomes belonging to various species, but also protein complements belonging to the whole taxonomic groups. We expect that the PHOG database will be useful in our efforts to understand such evolutionary phenomena as horizontal transfer, the existence of orphans genes, gene losses and gene acquisitions.

## Availability and requirements

The PHOG database is publicly accessible at  . The following browsers are recommended to access the web interface: Netscape 7.0 or higher, Internet Explorer 5.0 or higher.

## Authors' contributions

IM did all computation and wrote the manuscript. PN conceived this study and obtained the preliminary results. AM developed the approach for this research, provided overall guidance and revised this manuscript.
